# Genome Mining Reveals a Sactipeptide Biosynthetic Cluster in *Staphylococcus pseudintermedius*

**DOI:** 10.3390/vetsci12070635

**Published:** 2025-07-02

**Authors:** Ola K. Elsakhawy, Mohamed A. Abouelkhair

**Affiliations:** Diagnostic Medicine and Pathobiology, Shreiber School of Veterinary Medicine, Rowan University, 201 Mullica Hill Road, Glassboro, NJ 08028, USA

**Keywords:** antibiotics resistance, bacteriocin, subtilosin A, *Staphylococcus pseudintermedius*

## Abstract

*Staphylococcus pseudintermedius*, an opportunistic pathogen of veterinary and zoonotic concern, harbors diverse biosynthetic gene clusters (BGCs) that may contribute to its ecological fitness and virulence. In this study, we performed a comparative genomic analysis of 6815 *S. pseudintermedius* isolates. Using Roary, we identified core and accessory genomes, revealing the subtilosin A gene (*sboA*) as part of the accessory genome, present in a subset of *S. pseudintermedius* isolates from clinical (*n* = 657), environmental (*n* = 1031), and unclassified sources (*n* = 487). AntiSMASH v8.0.0 analysis confirmed the presence of subtilosin A BGCs annotated as a sactipeptide with low similarity confidence to *Bacillus subtilis subsp. spizizenii* ATCC 6633 subtilosin A cluster. Further characterization using BAGEL4 identified multiple genes homologous to the *Bacillus subtilis* subtilosin A biosynthetic machinery (*sbo-albABCDEFG*), although *albB*, *albG*, and *sboX* were not annotated, raising questions about cluster completeness and functionality. BLAST v2.12.0 analysis of the full BGC identified by BAGEL4, revealing high conservation (99.6–100% pairwise identity) of gene content and order in 395 clinical, 593 environmental, and 417 unclassified *S. pseudintermedius* isolates. Incomplete clusters were identified in 763 clinical, 942 environmental, and 201 unclassified *S. pseudintermedius* isolates. The discrepancy between the number of isolates containing *sboA* and those harboring the full cluster may reflect evolutionary divergence or could be attributed to limitations in assembly quality. The functional implications of the identified cluster in *S. pseudintermedius* remain to be elucidated; however, its potential role in conferring competitive fitness by inhibiting closely related species is supported by previous findings in other staphylococci.

## Simple Summary

*Staphylococcus pseudintermedius* is commonly found in dogs and is occasionally linked to infections in humans. The growing concern over *S. pseudintermedius* stems not only from its pathogenic potential but also from its rising resistance to multiple antibiotic classes. This resistance makes treatment more difficult and underscores the importance of identifying alternative strategies for managing infections. We conducted a comparative genomics analysis of *S. pseudintermedius* isolates. Interestingly, the genes similar to those coding for subtilisin A, a bacteriocin originally produced by *Bacillus subtilis*, were detected in *S. pseudintermedius* genomes. The core gene, *sboA*, was present in 657 *S. pseudintermedius* isolates from infected animals and humans, 1,031 *S. pseudintermedius* isolated from the environment, and 487 *S. pseudintermedius* isolates from unclassified sources, while the complete gene cluster was identified in only 395, 593, and 417 isolates, respectively. This discrepancy may reflect natural genetic variation or limitations in genome assembly quality. Our results show that *S. pseudintermedius* carries the genetic potential to produce subtilosin A, a bacteriocin known to kill a broad range of both gram-positive and gram-negative bacteria. These findings may support the development of new treatments that harness such natural antimicrobial compounds to help combat bacterial infections and address the growing challenge of antibiotic resistance.

## 1. Introduction

The rise of antibiotic-resistant bacterial infections has intensified the search for novel antimicrobial agents, including naturally occurring peptides known as bacteriocins. Bacteriocins are ribosomally synthesized antimicrobial peptides produced by bacteria to inhibit the growth of closely related or competing bacterial species. Among these, subtilosin A, a bacteriocin produced by *Bacillus subtilis* strain 168, has garnered considerable interest due to its broad-spectrum activity against both gram-positive and gram-negative bacteria, including *Enterococcus faecalis* OGX-1, *Listeria monocytogenes* ATCC 19115, *Porphyromonas gingivalis* ATCC 33277, *Kocuria rhizophila* ATCC 9341, *Enterobacter aerogenes* ATCC 13408, *Streptococcus pyogenes* ATCC 19615, and *Shigella sonnei* ATCC 25931 [1]. Subtilosin A belongs to a group of ribosomally synthesized and post-translationally modified peptides (RiPPs) called sactipeptides, which are characterized by sulfur-to-alpha carbon thioether cross-linked peptides. The biosynthesis of subtilosin is governed by the *sbo-albABCDEFG* gene cluster. The structural gene *sbo* encodes the subtilosin precursor peptide Sbo, which contains a leader peptide of eight residues and a core peptide of thirty-five residues. The radical S-adenosylmethionine (SAM) enzyme AlbA (an *albA* gene product), which contains a [4Fe-4S] cluster, catalyzes the formation of unique intramolecular thioether bonds of Sbo that crosslink the α-carbon of acceptor residues and the sulfur atom of cysteine residues. This thioether bond formation is dependent on the intact Sbo leader peptide sequence. The leader peptide is subsequently cleaved, and macrocyclization of the core peptide is mediated by AlbE and AlbF, encoded by the *albE* and *albF* genes. The final mature peptide is exported via the ABC transporter AlbC, encoded by the *albC* gene [2,3].

*Staphylococcus pseudintermedius* is primarily associated with companion animals, particularly dogs and cats, where it acts as both a commensal and an opportunistic pathogen. It is the most common cause of skin infections in dogs, including pyoderma, and can also cause wound infections, otitis, and post-surgical complications [4]. Though historically considered an animal-specific pathogen, *S. pseudintermedius* has increasingly been recognized as a zoonotic agent capable of causing infections in humans, particularly in those with close contact with pets [5,6,7,8]. The growing concern over *S. pseudintermedius* stems not only from its pathogenic potential but also from its rising resistance to multiple antibiotic classes, including β-lactams and macrolides [9,10,11,12,13]. This resistance complicates treatment and underscores the importance of identifying alternative strategies for managing infections.

To better understand the genetic factors that may contribute to its antimicrobial capabilities and pathogenicity, we conducted a comparative genomics analysis of *S. pseudintermedius* isolates. Interestingly, the genes similar to those coding for subtilisin A, a bacteriocin originally produced by *Bacillus subtilis*, were detected in *S. pseudintermedius* genomes. Our findings suggest the potential for subtilosin production in this species.

## 2. Materials and Methods

### 2.1. Isolates Included in the Study

We used the National Center for Biotechnology Information (NCBI) Pathogen Detection system (BETA) (https://www.ncbi.nlm.nih.gov/pathogens/, accessed in January 2025) to obtain *Staphylococcus pseudintermedius* genome sequences for this study. This integrated system compiles bacterial and fungal genomic data from ongoing surveillance and research efforts, including isolates obtained from food, environmental sources, and clinical samples from patients. The NCBI Pathogen Detection system clusters related pathogen genome sequences to help identify potential transmission chains, supporting outbreak investigations by public health officials, and screens genome sequences as part of the National Database of Antibiotic Resistant Organisms (NDARO) using the AMRFinderPlus tool [14]. This allows for the detection of genes coding for antimicrobial resistance, stress response, and virulence proteins, providing insight into the spread and evolution of resistance and pathogenicity.

We filtered S. pseudintermedius isolates by source, including those isolated from clinical, environmental, or unclassified sources, to ensure that both infection-related and putative environmental reservoirs were adequately represented.

### 2.2. Genome Mining by antiSMASH and BAGEL4

To identify biosynthetic gene clusters (BGCs) associated with secondary metabolite production, we employed antiSMASH v8.0 (https://antismash.secondarymetabolites.org) [15] and BAGEL4 (http://bagel4.molgenrug.nl/) [16]—two widely used web-based platforms for genome mining. AntiSMASH analyzes bacterial genomes for potential BGCs by comparing gene architectures with a curated database of known biosynthetic pathways, while BAGEL4 is tailored to detect bacteriocins and ribosomally synthesized and post-translationally modified peptides (RiPPs) in bacterial (meta-) genomic DNA. The default parameters were applied, and only high-quality assemblies were included to ensure accurate annotation and characterization of candidate clusters.

### 2.3. Sequence Analysis

All sequence analyses were conducted using Geneious Prime^®^ 2025.1.3 software [17], which integrates a variety of bioinformatics tools. Multiple sequence alignments were performed using Clustal Omega v1.2.3. For homology and identity searches, we generated and queried a custom BLAST database within Geneious Prime^®^ 2025 software to ensure accurate comparison against genome sequences from reference, clinical, and environmental *S. pseudintermedius* isolates and those isolated from unclassified sources. For the pangenomic analysis, *S. pseudintermedius* genome assemblies were downloaded from NCBI (https://www.ncbi.nlm.nih.gov/). The assemblies were subjected to prokka v1.14.5 annotation (https://github.com/tseemann/prokka) [18] and the Roary v3.13.0 pangenome pipeline (https://github.com/sanger-pathogens/Roary) [19]. The default parameters were applied.

## 3. Results

**Figure 1:**
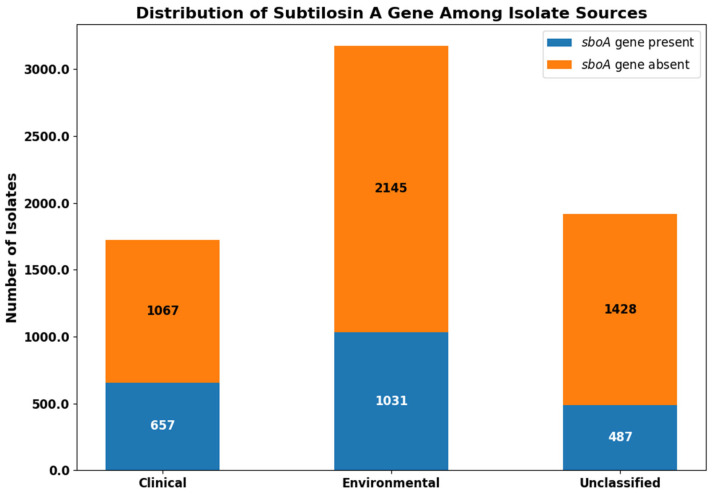
Distribution of the subtilosin A core gene (*sboA*) among *Staphylococcus pseudintermedius* isolates. The *sboA* gene was detected in 657 of 1724 clinical isolates, 1031 of 3176 environmental isolates, and 487 of 1915 unclassified isolates. Bar segments represent the number of isolates present (blue) or absent (orange) for the *sboA* gene in each source category.

A total of 6815 *S. pseudintermedius* isolates were retrieved from the NCBI Pathogen Detection database. We performed pan-genome analysis using Roary, which enabled the identification of core and accessory genes among the genomes. Through this analysis, we observed that the subtilosin A core gene (*sboA*) was present in 657 of 1724 clinical isolates (38.1%), 1031 of 3176 environmental isolates (32.5%), and 487 of 1915 unclassified isolates (25.4%) Figure 1.

**Figure 2:**
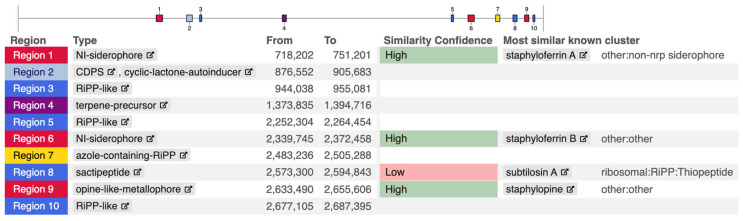
Biosynthetic gene clusters identified in *Staphylococcus pseudintermedius* 081661 using antiSMASHV8.0.0.

To explore whether the entire subtilosin A secondary metabolite biosynthetic gene cluster (BGC) was present, the complete genome of *S. pseudintermedius* isolates was analyzed using antiSMASH version 8.0.0 [15]. The tool was run with default parameters, allowing for the detection of a wide range of BGC types. Each cluster’s similarity to known BGCs was evaluated using the built-in similarity comparison module. The number and types of BGCs varied across genomes, reflecting strain-specific biosynthetic diversity. *S. speudintermedius* 081661 was selected for detailed visualization, as shown in Figure 2. Notably, region 8 in this isolate was identified as a sactipeptide with low similarity confidence to the known and well-characterized subtilosin A cluster in the database. The low similarity confidence reflects limited homology to characterized clusters, suggesting either sequence divergence or novel gene architecture.

**Figure 3:**
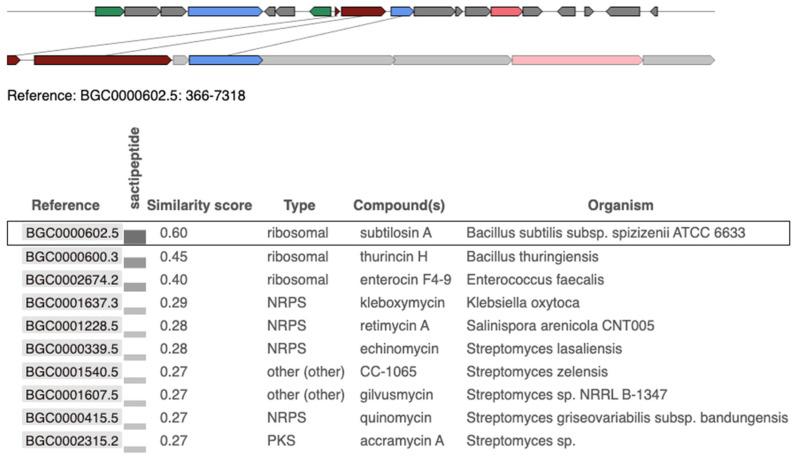
Comparison of the subtilosin A biosynthetic gene cluster (BGC) identified in *Staphylococcus pseudintermedius* 081661 with reference BGCs from the MIBiG database using antiSMASHV8.0.0 analysis. The top panel shows a visual representation of the query region from *S. pseudintermedius* (bottom track) aligned with the best-matching reference cluster, subtilosin A from *Bacillus subtilis subsp. spizizenii* ATCC 6633 (top track). Genes are represented as arrows, with lines connecting orthologous genes based on the best 1:1 BLAST matches. The lower panel shows the genetic similarity analysis of the identified subtilosin A BGC from *S. pseudintermedius 081661*. The highlighted row (black border) indicates the currently selected match, subtilosin A (BGC0000615), which shares 60% similarity with the query cluster and is classified as a ribosomal sactipeptide cluster. Red: core biosynthetic genes; Orange: additional biosynthetic genes; Blue: transport-related genes; Green: regulatory genes; Gray: other or uncharacterized genes.

The genetic similarity and organization of the BGCs were analyzed using the “Minimum Information about a Biosynthetic Gene Cluster” (MIBiG) database. The BGC from *S. pseudintermedius 081661* showed a similarity score of 0.60 with subtilosin A-producing *Bacillus subtilis* subsp. *spizizenii* ATCC 6633 Figure 3. A similarity score of 0.45 was observed with a thurincin H-producing *Bacillus thuringiensis*, and a score of 0.40 was linked to an enterocin F4-9-producing *Enterococcus faecalis* Figure 3.

To further validate and refine these findings, the BAGEL4 web server was utilized to mine *S. pseudintermedius* genome assemblies for bacteriocins and ribosomally synthesized and post-translationally modified peptides (RiPPs). BAGEL4 analysis helped to better characterize the components of the subtilosin A biosynthetic gene cluster, providing additional insights into gene content. BAGEL4 analysis of *S. pseudintermedius* genome assemblies confirmed the presence of a biosynthetic cluster associated with subtilosin A biosynthesis in some *S. pseudintermedius* isolates. The cluster was annotated as Subtilosin A and included several hallmark genes involved in sactipeptide biosynthesis. Key components identified within the cluster included *albA* (encoding an antilisterial bacteriocin subtilosin biosynthesis protein albA; BmbF), *albD* (encoding an antilisterial bacteriocin subtilosin biosynthesis protein albD; orf00020), *albE* (encoding an antilisterial bacteriocin subtilosin biosynthesis protein albE; orf00023), and albF, the latter annotated as a putative *zinc-dependent protease* (OS = Bacillus subtilis, GN = albF). An ABC transporter protein (ALBC_BACSU, PF00005) was also present, indicating a potential role in peptide export or immunity. However, *albB*, *albG*, and *sboX* were not annotated/detected.

Additional open reading frames (ORFs), such as orf00022 and orf00025, remained functionally uncharacterized, suggesting possible novel or accessory functions within the identified subtilosin cluster. The detection of these genes in several isolates supports the hypothesis of a conserved, yet potentially divergent, subtilosin A bacteriocin system in *S. pseudintermedius*.

**Figure 4:**
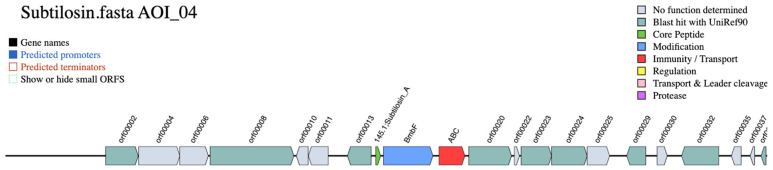
Genetic organization of the subtilosin A biosynthetic gene cluster identified by BAGEL4 in *Staphylococcus pseudintermedius* 081661. The cluster was visualized with predicted functions color-coded according to gene classification. The core peptide (green), the protein involved in the modification process (blue), and transport (red).

The overall genetic architecture of the subtilosin A gene cluster in *S. pseudintermedius 081661* is illustrated in Figure 4.

**Figure 5:**
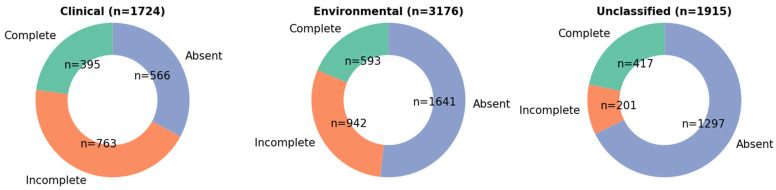
The distribution of *Staphylococcus pseudintermedius* isolates containing subtilosin A genes across three sources: clinical, environmental, and unclassified. Each pie chart displays the proportion of isolates with complete, incomplete, or absent subtilosin A gene clusters. Complete: Isolates with full subtilosin A gene cluster; Incomplete: Isolates with partial/subset of subtilosin A genes; Absent: No detectable subtilosin A gene components. Colors: greenish = #66c2a5, orange = #fc8d62, blueish = #8da0cb.

To assess the conservation and synteny of the subtilosin A biosynthetic gene cluster (BGC), we performed a BLAST analysis of the entire cluster identified by BAGEL4 against all downloaded *S. pseudintermedius* genomes. This analysis revealed that the full cluster, including gene content and order, was conserved in a subset of the genomes. Specifically, the subtilosin A biosynthetic gene cluster was detected in 395 clinical isolates, with sequence lengths ranging from 6315 to 6325 bps and nucleotide identity of 99.6–100% to the *S. pseudintermedius* 081661 subtilosin A BGC Figure 5. Additionally, 593 isolates from environmental sources harbored the cluster with lengths of 6316 to 6328 bps and nucleotide identity of 99.6–99.98% to the *S. pseudintermedius* 081661 subtilosin A BGC. Furthermore, 417 isolates from unclassified sources carried the cluster with lengths of 6316 to 6326 bps and nucleotide identity of 99.6-99.98% to the *S. pseudintermedius* 081661 subtilosin A BGC Figure 5. These findings demonstrate the widespread but not universal conservation of the subtilosin A cluster among *S. pseudintermedius* isolates from clinical, environmental, and unclassified sources Figure 5. Importantly, incomplete or partial subtilosin A clusters were observed in a greater number of genomes. These truncated clusters may reflect technical limitations, such as incomplete genome assemblies, fragmented contigs, or variations in sequencing technology, depth, and quality. Alternatively, they could indicate evolutionary differences in the BGC architecture.

## 4. Discussion

In this study, we focused on *Staphylococcus pseudintermedius*, an opportunistic pathogen that primarily colonizes the skin and mucosal surfaces of dogs and other companion animals. It is increasingly recognized as a significant cause of veterinary infections, including pyoderma, otitis, and postoperative wound infections [20]. Moreover, *S. pseudintermedius* has zoonotic potential, with reports of transmission to humans, particularly among pet owners and veterinary personnel. The clinical relevance of this study is underscored by the growing global concern surrounding *methicillin-resistant Staphylococcus pseudintermedius* (MRSP). Currently, MRSP presents a veterinary health challenge comparable to the public health crisis posed by *methicillin-resistant Staphylococcus aureus* (MRSA) in human medicine. The increasing spread of multidrug-resistant MRSP and methicillin-susceptible *S. pseudintermedius* (MSSP) strains, coupled with the lack of effective new antibiotics, highlights the limited therapeutic possibilities and the urgent need for alternative strategies such as bacteriocin-based interventions to control and treat infections [1,21].

Among the bacteriocins, subtilosin A—a ribosomally synthesized and post-translationally modified peptide (RiPP)—is known for its broad-spectrum antimicrobial activity, particularly against Gram-positive bacteria [22]. The biosynthesis of subtilosin is governed by the *sbo-albABCDEFG* gene cluster. Our analysis revealed that the subtilosin A biosynthetic gene cluster (BGC) is conserved in a subset of S. pseudintermedius isolates. Notably, the presence of key genes coding for subtilosin A (sboA), albA, albD, albE, and albF, along with an associated ABC transporter, suggests a functional sactipeptide biosynthetic pathway. However, we also observed incomplete or partial clusters in a greater number of genomes, which may be attributed to technical limitations like incomplete genome assemblies or sequencing technology and quality. Alternatively, these variations could reflect evolutionary differences in the BGC architecture. Importantly, these observations emphasize the need for experimental validation. Techniques such as RNA-seq and proteomics are essential to confirm not only the transcription and translation of these genes but also the actual production of subtilosin A in vitro and its functional role as a bacteriocin.

A recently published comprehensive population and pan-genomic study of *Staphylococcus pseudintermedius* by Zehr et al., which we came across during manuscript submission, supports our findings [23].

A previous study has identified the subtilosin A BGC in *Staphylococcus felis and Staphylococcus delphini* [2]. The identified cluster in *S. felis* included Sbo, AlbA, AlbC, AlbD, AlbE, AlbF, and AlbG, but lacked AlbB, whereas in *S. delphini*, the cluster included Sbo, AlbA, AlbB, AlbC, AlbD, AlbE, and AlbF, but lacked AlbG [2].

Zheng et al. demonstrated that *Bacillus subtilis* mutants lacking *albE* and *albF* exhibited reduced or abolished subtilosin production, highlighting the essential role of these genes in subtilosin maturation [24]. Intriguingly, our bioinformatic analysis revealed that *albB*, *albG*, and *sboX* are absent/not annotated in all examined clinical and environmental isolates of *Staphylococcus pseudintermedius* and even those from unclassified sources. This raises important questions about whether *S. pseudintermedius* employs an alternative mechanism for subtilosin processing or produces a structurally distinct, functionally analogous molecule. Further transcriptomic and proteomic analyses are warranted to determine whether subtilosin or a related compound is synthesized and matured through a different pathway in this species.

Interestingly, the presence of subtilosin A biosynthetic gene cluster (BGC) in *S. pseudintermedius* adds to a growing body of evidence suggesting that staphylococci possess diverse and potentially bioactive bacteriocin gene clusters [25,26,27,28]. For instance, *Staphylococcus hyicus* 4244 was recently reported to produce a novel sactibiotic, hyicin 4244, the first of its kind identified in staphylococci. This bacteriocin exhibits a broad spectrum of antimicrobial activity, including efficacy against multidrug-resistant staphylococcal strains, and has demonstrated the ability to prevent biofilm formation and reduce established biofilms on abiotic surfaces in vitro [29]. These findings highlight the biotechnological potential of sactibiotics not only in therapeutic settings but also in the development of antimicrobial coatings for medical devices.

The widespread presence of the subtilosin A BGC in a substantial subset of *Staphylococcus pseudintermedius* isolates, spanning both clinical and environmental origins, suggests that this cluster may play an important role in the ecological success of this species.

In microbial ecosystems, species often compete for limited resources such as nutrients, colonization sites, and ecological space. One common strategy for gaining a competitive advantage involves the secretion of antimicrobial compounds, including bacteriocins, which target closely related species [30,31,32,33,34,35]. The identification of subtilosin A gene clusters in *S. pseudintermedius* suggests this bacterium may utilize such a strategy to enhance its ecological success. If functionally expressed, subtilosin A could enable *S. pseudintermedius* to inhibit the growth of competing skin-associated microbes, thereby promoting its persistence on host surfaces and increasing its colonization efficiency [35,36]. This is particularly relevant in polymicrobial environments, where subtle shifts in microbial population dynamics can determine pathogenic potential or persistence. The conservation of the subtilosin A cluster in many *S. pseudintermedius* genomes underscores its likely benefit; however, the absence of key genes in some genomes, as well as the lack of annotation for potential regulatory elements such as *sboX*, raises questions about whether the cluster is universally functional in these strains.

Ultimately, the detection of these clusters at the genomic level does not confirm functional expression. To determine whether the subtilosin A BGC is actively transcribed and translated into a functional antimicrobial product, further studies incorporating RNA-seq, proteomics, and biochemical assays are needed [37,38]. These approaches will be critical for verifying bacteriocin production and understanding its regulatory mechanisms, as well as elucidating its role in inter-bacterial interactions and potential contribution to virulence or colonization. Confirming the functional activity of subtilosin A in *S. pseudintermedius* may also open new avenues for exploring its therapeutic potential as a targeted antimicrobial agent.

## 5. Conclusions

In conclusion, our genomic analysis of *S. pseudintermedius* revealed the widespread presence and conservation of the subtilosin A biosynthetic gene cluster in both clinical and environmental isolates. Although some genomes contained incomplete clusters, likely due to sequencing quality or evolutionary divergence, the core components were consistently identified in a significant subset of isolates. The findings suggest that subtilosin A, if functionally expressed, may contribute to the competitive fitness of *S. pseudintermedius* in complex microbial environments. However, the absence of key genes in some isolates and the lack of annotation for regulatory elements such as *sboX* highlight the need for further experimental validation. Future work integrating transcriptomic and proteomic approaches is essential to confirm subtilosin A expression and elucidate its functional role in bacterial competition and pathogenesis.

## Data Availability

The original contributions presented in this study are included in the article. Further inquiries can be directed to the corresponding author.
